# Informing the UK Muslim Community on Organ Donation: Evaluating the Effect of a National Public Health Programme by Health Professionals and Faith Leaders

**DOI:** 10.1007/s10943-022-01680-9

**Published:** 2022-10-07

**Authors:** Omar M. E. Ali, Eleftherios Gkekas, Ahmad M. S. Ali, Tsz Yau Tiffany Tang, Sameer Ahmed, Imadul Chowdhury, Salman Waqar, Amer Hamed, Sharif Al-Ghazal, Saeed Ahmed

**Affiliations:** 1 Newcastle University Medical School, Newcastle upon Tyne, NE2 4HH UK; 2 Department of Neurosurgery, The Walton Centre for Neurology and Neurosurgery, Liverpool, L9 7LJ UK; 3 Renal Unit, South Tyneside and Sunderland NHS Foundation Trust, Kayl Rd, Sunderland, SR4 7TP UK; 4 Royal Victoria Infirmary, Newcastle Upon Tyne Hospitals NHS Foundation Trust, Queen Victoria Rd, Newcastle upon Tyne, NE1 4LP UK; 5 Brighton and Sussex Medical School, University of Sussex Brighton, East Sussex, BN1 9PX UK; 6 Nuffield Department of Primary Care Health Sciences, University of Oxford, Woodstock Rd, Oxford, OX2 6GG UK; 7 Stepping Hill Hospital, Stockport NHS Foundation Trust, Poplar Grove, Hazel Grove, Stockport, SK2 7JE UK; 8 Bradford Teaching Hospitals NHS Foundation Trust, Bradford, BD9 6RJ UK

**Keywords:** Organ donation, Transplantation, Muslim, Black, Asian and minority ethnic (BAME), Inequalities, Faith, United Kingdom

## Abstract

There is a significant shortage of transplantable organs in the UK particularly from Black, Asian and Minority Ethnic (BAME) groups, of which Muslims make a large proportion. The British Islamic Medical Association (BIMA) held a nationwide series of community gatherings with the aim of describing the beliefs and attitudes to organ donation amongst British Muslims and evaluate the efficacy of a national public health programme on views and uncertainties regarding religious permissibility and willingness to register. Eight public forums were held across the UK between June 2019 and March 2020 by the British Islamic Medical Association (BIMA). A panel of experts consisting of health professionals and Imams discussed with audiences the procedures, experiences and Islamic ethico-legal rulings on organ donation. Attendees completed a self-administered questionnaire which captured demographic data along with opinions before and after the session regarding religious permissibility and willingness to register given permissibility. A total of 554 respondents across seven UK cities were included with a M:F ratio 1:1.1. Only 45 (8%) respondents were registered as organ donors. Amongst those not registered multiple justifications were detailed, foremost of which was religious uncertainty (73%). Pre-intervention results indicated 50% of respondents were unsure of the permissibility of organ donation in Islam. Of those initially unsure or against permissibility or willingness to register, 72% changed their opinion towards deeming it permissible and 60% towards a willingness to register indicating a significant change in opinion (*p* < 0.001). The effectiveness of our interventions suggests further education incorporating faith leaders alongside local healthcare professionals to address religious and cultural concerns can reduce uncertainty whilst improving organ donation rates among the Muslim community.

## Introduction

There is a significant shortage of organ donors from Black, Asian and Minority Ethnic (BAME) groups in the United Kingdom (UK) despite numerous public education campaigns. BAME groups represent 14% of the British population, (Office for National Statistics, 2011a) but only 7% of the opt-in NHS Organ Donation Register, and 31% of patients on the transplant waiting list (NHS Blood and Transplant, 2019). There is therefore a large discrepancy between need for transplantation and availability of well-matched organs, with specific blood group and tissue type combinations more common among minority ethnic groups. Median waiting times for adult kidney transplants for BAME patients are approximately 230 days longer than for White patients (NHS Blood and Transplant, 2019). This has implications on survival and quality of life, as well as increasing costs to the NHS given longer waiting times and most patients receiving dialysis as an alternative form of renal replacement therapy.

Barriers to organ donation (OD) reported by members of the BAME community include a lack of knowledge on the process of OD and registration, faith and cultural beliefs, bodily concerns with regards to disfigurement and resurrection, family influence and a mistrust in doctors and the healthcare system (AlKhawari et al., 2005; Morgan, 2015).

Muslims represent 5% of the British population (Office for National Statistics, 2011b) and an estimated one-third of the UK BAME population (Muslim Council of Britain, 2015). Many Muslims perceive the position of their religion as a decisive factor in their behaviour towards OD (Ghaly, 2012). The majority, though not all, of Islamic scholarly opinion is in favour of organ donation’s permissibility—in 1995, the Muslim Law (Shariah) Council of the UK issued a fatwa (legal verdict) deeming OD permissible, in line with major global religious institutions such as the European Council for Fatwa and Research, Islamic Fiqh Academy of the Organisation of Islam Conference, and Al-Azhar Academy of Egypt (Ryan, 1996). These legal verdicts are non-binding, and Muslims are free to select any appropriately issued fatwa based on the presented arguments and perceived moral authority of the jurisconsult. However, a lack of familiarity with religious rulings has been consistently demonstrated amongst Muslim communities in the UK and abroad (Afzal Aghaee et al., 2015; Ahmed et al., 1999; Altraif et al., 2020; Aslam & Hameed, 2008). Multiple studies have correlated increased awareness of the prevalence of organ donation and its Islamic opinion with increasing willingness to donate (Afzal Aghaee et al., 2015; Al Moweshy et al., 2022; Krupic et al., 2019; Taş et al., 2021).

With the recent law change in the UK and transition to an opt-out system, it is important to enable British Muslim communities to make fully informed decisions. Preliminary ethnicity data suggests those who opt-out of the donor register are more likely to be from BAME background, and 56% of opt-outs in 2019 were made by people of an Asian background (NHS Blood & Transplant, 2019). The UK's OD Taskforce recognised an urgent need to identify and implement the most effective methods to promote OD and registration to the public generally and ethnic minority populations specifically (Department of Health, 2008). Multiple public health educational methods have been explored in the literature (Baines et al., 2002; Morgan et al., 2016). Mass media campaigns have shown limited effect in producing change at a registration level and dealing with the varied concerns of minority groups (Oliver et al., 2010; Deedat et al., 2013; Khan & Randhawa, 1999). A strong interpersonal component increases success rates, and the previous use of “peer educators”– ordinary members of the community belonging to minority groups–in holding OD awareness events has proven successful (Krupic et al., 2019; Long et al., 2013).

Engaging faith leaders has been shown to be of value in facilitating health behavioural changes amongst their respective communities. Local faith leaders act as “boundary spanners” who can permeate organisational and cultural boundaries and be a bridge between organisations and local communities (Long et al., 2013). Extensive involvement of local and regional faith institutions was observed during the coronavirus pandemic in promoting precautionary measures and vaccination amongst communities typically considered hard to reach (Dascalu et al., 2021; Gildea, 2021; Guthrie et al., 2021; Randhawa et al., 2010; Wells et al., 2022). For example, healthcare chaplains have been noted to regularly engage in organ procurement discussions, and their involvement in providing training for other community Imams in navigating these topics should be encouraged (Carey et al. 2011).

The primary aim of this study is to examine the effect of an educational session delivered by local healthcare professionals and religious leaders within different Muslim communities on levels of awareness and uncertainty pertaining to the process and Islamic discussion on OD. The secondary aims of this study were to explore both the effect of these interventions in resolving the uncertainties among attendees unsure of the Islamic ethico-legal attitudes towards OD and the willingness of these communities to register for OD post-intervention.

Our first hypothesis was that there is a substantial lack of awareness of or familiarity with established religious rulings on OD amongst British Muslims affecting willingness to register. Our second hypothesis was that a focused educational intervention delivered by Imams and health professionals informing Muslims of the current OD process and Islamic legal discourse can increase willingness to register.

## Methods

The British Islamic Medical Association (BIMA) organised a national campaign named “Let’s Talk about Organ Donation” with the aim of determining British Muslims’ attitudes towards OD and increasing awareness of the OD process (Ali et al., 2020b). Between June 2019 and March 2020, eight open public forums were conducted across the country (sittings in Glasgow, Leeds, London, Manchester, Newcastle, Nottingham and two in Bradford). These locations were selected for their relatively generous local Muslim populations. The events were held in a mixture of settings including public spaces, mosques and universities. Events were advertised to the local community via social media, mosque announcements, the distribution of posters and leaflets in mosques and Islamic study circles, GP surgeries, pharmacies and through word of mouth. The first session was a pilot run in Newcastle with 86 subjects to evaluate and receive feedback on feasibility, session content and the questionnaire (Ali et al., 2020a).

During each event, attendees listened to a panel of experts consisting of various OD and transplantation healthcare professionals, specialist nurses in OD, Islamic scholars, local Imams and Muslim patients who had received or were waiting for an organ transplant. Healthcare professionals included consultants in Critical Care, Nephrology, Transplant surgery and regional clinical leads for OD. Whilst certain sections such as the Islamic bio-ethical discussion were delivered by the same speaker and the same presentation slides were used, other healthcare speakers were local and therefore varied with location. The same range of speaker types was pursued as above to promote consistency in messaging. Panel speakers were identified by a central BIMA OD team in collaboration with local BIMA teams. The purpose of the campaign was explained and content from the pilot event shared to highlight the main themes covered and to act as a framework for the forum’s targets and learning objectives. Speakers were provided advice in advance on emphasising the impartiality of content delivery and an understanding of the accepted heterogeneity in acceptable scholarly opinion. The panel was succeeded by a live Q&A session.

Each intervention lasted approximately 2–3 hours and involved discussions on the following topics: the panel introducing the concept of OD and relevant statistics, patient experiences of being a recipient or on the waiting list, the OD process and law change, a discussion on British Muslims’ attitudes and organ transplantation from the perspective of the Shariah (Islamic law). The latter discussion involved familiarising the audience with the current available fatawa on OD, the ethical and moral discourse behind scholars’ conclusions and addressing common misconceptions about OD.

Attendees were asked to complete a 9-item anonymised, confidential, self-administered questionnaire in English comprising mostly closed-ended questions with specific answer categories. Due to time constraints in delivering this intervention before the UK system change, our questionnaire was not validated. The questionnaire was composed of two sections (Appendix 1). Part A included questions about age, gender, ethnicity, OD registry status and whether the attendee had ever considered joining the registry and if the answer was negative, an open question regarding the reasoning behind this. Subsequently, there were two questions which were each repeated in part A (before the panel discussion) and part B (after the panel discussion). These questions asked whether the attendee felt OD is permissible, and given permissibility whether they would register for OD.

Results on the categorical variables were presented as percentage values. Analysis was performed using version 26 of the SPSS software. Pearson’s Chi-squared statistical test was used to evaluate correlations between different variables. Values with *p* < 0.05 were deemed statistically significant.

## Results

### Demographics

A total of 554 attendees completed the questionnaire. The total number of attendees was not recorded due to the open nature of the forums.

Respondents were subdivided into groups based on age, gender, ethnic origin, location, and OD card possession. Respondent demographics are highlighted in Table 1. The male to female ratio was 1:1.1. The most prevalent ethnic group within the study cohort was Pakistani (57.4%), followed by Indian (12.5%) and Arab (9.2%). Bradford (30.3%) had the highest proportion of participants across its two sittings, followed by Nottingham (18.2%) and Newcastle (15.5%).

**Table 1:** Demographic data

Demographics	Number of respondents (%)
*Age (years)*	
< 20	98 (**17.7**)
21–40	219 (**39.5**)
41–60	182 (**32.9**)
61–80	52 (**9.4**)
> 80	3 (**0.5**)
*Gender*	
Male	266 (**48.0**)
Female	288 (**52.0**)
*Ethnicity*	
Pakistani	318 (**57.4**)
Indian	69 (**12.5**)
Arab	51 (**9.2**)
Bangladeshi	49 (**8.8**)
White	29 (**5.2**)
Other	38 (**6.9**)
*Location*	
Bradford	168 (**30.3**)
Glasgow	36 (**6.0**)
Leeds	54 (**9.7**)
London	58 (**10.4**)
Manchester	51 (**9.2**)
Newcastle	86 (**15.5**)
Nottingham	101 (**18.2**)

### Organ Donation Registration

Only 45 (8.1%) respondents were already registered for OD before the event, and of those not registered, 138 (27.1%) indicated they had previously thought about registering. Those who documented their reasons for not registering (*n* = 127) cited multiple reasons broadly classified as faith beliefs and views on religious permissibility (73%), lack of knowledge on OD (21%), family influence and reluctance to discuss OD (2%), death and burial concerns (2%) and moral considerations (2%). Though the sample size of white participants was small, respondents from BAME backgrounds (Pakistani, Indian, Arab, Bangladeshi) were significantly less likely to be registered as organ donors than their White counterparts (*p* < 0.001), with 10 out of 29 (34.5%) White ethnicity respondents already registered but only 33 out of 487 (6.8%) respondents of BAME background (Ali, 2020).

### Responses to Question 1 and 2

**Fig. 1:**
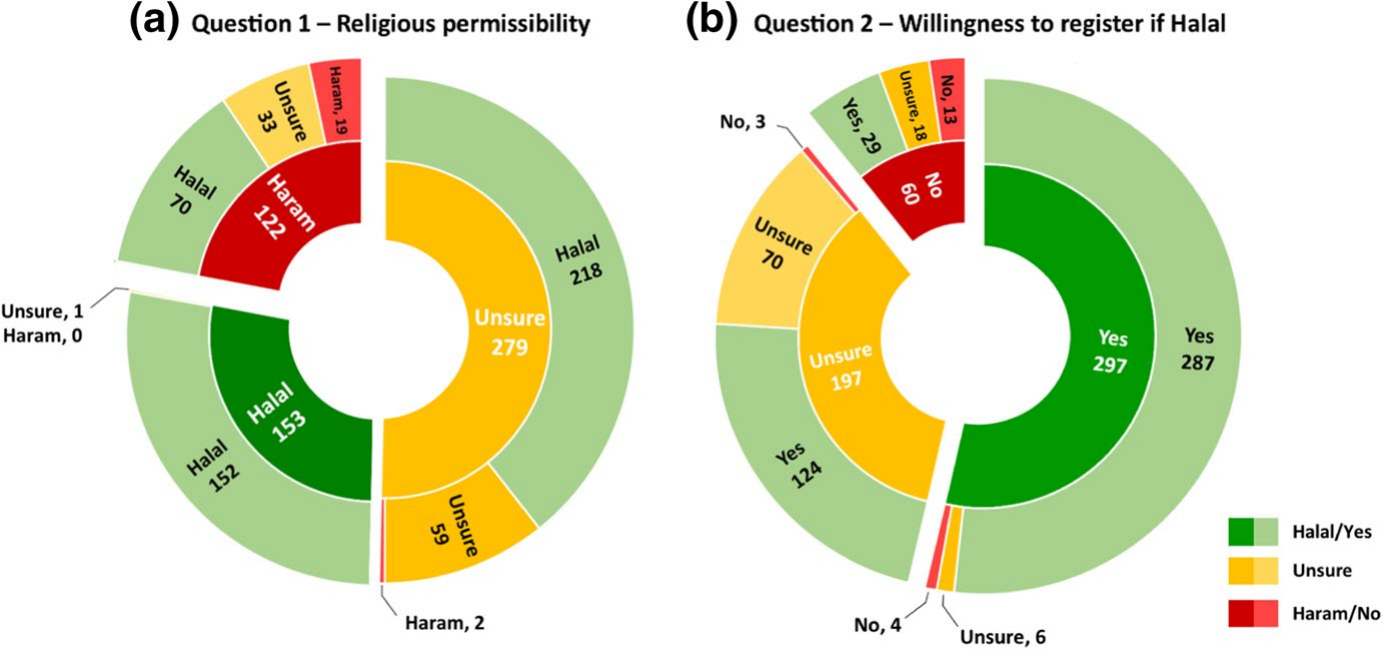
Pre-and post-intervention perceptions of **a** religious permissibility, **b** willingness to register if OD was considered Halal. Inner ring displays the number of responses pre-intervention. Outer ring displays the number of responses post-intervention, broken down according to pre-interventional response

Before the education session, when questioned on their perception of the permissibility of OD in Islam (Question 1), a minority considered OD to be permissible (27.6%) and half (50.4%) were unsure. After the education session, 79.4% of participants considered OD permissible, amounting to a 51.8% increase (*p* < 0.001, see Fig. 1). There was a corresponding 18.2% decrease in participants deeming OD impermissible (*p* < 0.001), coupled with a reduction of 33.6% in participants among the ‘Unsure’ population (*p* = 0.006).

**Fig. 2:**
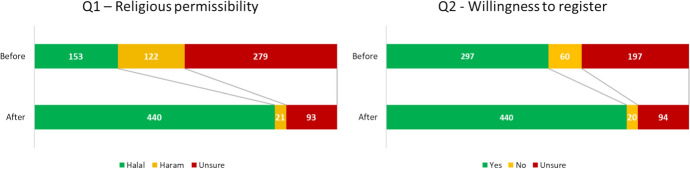
Stacked chart demonstrating overall change in number of responses for Questions 1 and 2 before and after the intervention

Question 2 explored whether the respondent would consider registering as an organ donor under the condition that OD was religiously permissible (Question 2). A total of 53.6% of participants answered ‘Yes’ pre-intervention; the remaining 46.4% answered ‘No’ or ‘Unsure’. Post-intervention there was a 25.8% increase in participants answering ‘Yes’ (*p* < 0.001), coupled with a decrease of 7.2 and 18.6% in respondents objecting to or unsure of registration, respectively (*p* < 0.001, see Fig. 2).

### Statistical Analysis

For Question 1 no specific age or gender group was more likely to select a particular response pre-or post-intervention, and generally most groups manifested a significant shift post-intervention towards permissibility and away from impermissibility. Across all ethnic groups evaluated, a statistically significant increase in ‘permissible’ responses and decrease in ‘impermissible’ responses following the intervention was observed using the Chi-squared test. The Pakistani subgroup had the greatest proportion of ‘impermissible’ responses pre-intervention at 25.8% of responses, which became 4.4% post-intervention, demonstrating a statistically significant 21.4% reduction (*p* < 0.001). With regards ‘unsure’ responses, only the Arab subgroup demonstrated a significant reduction post-intervention (*p* = 0.024). Subjects who did not possess an OD card were mostly ‘unsure’ before the intervention (52.5%). Within the subgroup not possessing an OD card, all shifts of opinion were statistically significant. No statistically significant changes were found in the smaller subgroup already in possession of an OD card. Within the group already in possession of an OD card, 12 (26.7%) thought OD was ‘impermissible’ pre-intervention.

For Question 2, a statistically significant increase in “Yes” responses and decrease in “Unsure” responses was observed across all age groups, with all groups under 55 years also showing a significant decrease in “No” responses (*p* < 0.05). Across both sexes, all such net shifts were statistically significant. There was a statistically significant increase in ‘Yes’ responses for all ethnicities (*p* < 0.05), except for respondents of white ethnicity. All but respondents of Arab background showed a statistically significant decrease in “No” responses, the largest of which was amongst Pakistanis at 8.2% (*p* < 0.001). All ethnicities demonstrated a statistically significant fall in the ‘Unsure’ responses, with the white population having the largest decrease at 31.0% (*p* = 0.046). There appeared to be a general post-interventional decrease in ‘No’ and ‘Unsure’ answers regardless of possession of an OD card. Consequently, there was a corresponding increase in ‘Yes’ responses towards Question 2, which is a pattern observed in all previous subgroups.

## Discussion

This study aims to explore the effects of an educational intervention aimed at Muslim communities around the UK delivered by healthcare professionals alongside local Imams and scholars, on perceptions towards religious permissibility for OD and willingness to register as a donor.

Our findings suggest a consistent post-interventional increase in the number of attendees considering OD religiously permissible (Figs. 1, 2). This trend presents in tandem with a post-interventional decrease in participants considering OD impermissible, were unwilling to register as a donor or were unsure of either. Figure 2 demonstrates the general shift in opinion post-intervention.

With regards to the first hypothesis pertaining the effect of religious uncertainty on willingness to register, it was found that most respondents were indeed unaware of the religious position on the matter, and religious concerns were cited as the foremost constraint amongst 73% of respondents. A strong emphasis on understanding Islam’s position has been found in multiple studies (Tarabeih et al., 2022; Altraif et al., 2020). Compared to 1% of White families, 30% of Asian families cite religious beliefs as reasons for refusing to consent for OD (NHS Blood & Transplant, 2019). Amongst South Asian faith groups, Muslims demonstrated significantly less favourable attitudes to OD than their Sikh and Hindu contemporaries, with religious guidance deemed more influential to Muslims’ decision-making (Karim et al., 2013). A global survey found that 69% of Muslims living in the West agreed with OD in principle but only 39% deemed it compatible with their religion (Sharif et al., 2011). A small proportion of our cohort did indeed believe OD was impermissible and were unwilling to register pre- and post-intervention, and a much smaller proportion shifted towards an objection of OD post-intervention. Although this is a valid and respected opinion within the corpus of Muslim scholarship and is to be accepted in the process of attaining informed consent, our study identified a much larger proportion of Muslims inclined initially towards uncertainty, the majority of whom shifted post-intervention towards an approval of OD. The results of this study corroborate the feasibility and positive impact of incorporating faith leaders in tackling religiously informed barriers.

Religiously tailored interventions can influence health behaviours at multiple levels of socio-ecological change due to the religion’s influence on individual, social, organisational and environmental aspects of people’s lives (Pratt et al., 2020). From the perspective of community-based participatory research (CBPR), health promotion programs that address the multidimensional nature of health problems may be more complex to implement but are more likely to result in lasting behavioural change (Campbell et al., 2007). Involving local Imams and using local mosques can be an effective method to deliver health-based interventions to populations that are sometimes considered marginalised, hard to reach or who view traditional health care channels with distrust. Their involvement can help bring home controversial topics such as organ donation when community members observe such discussions occurring in the familiar environment of their local mosque or openly addressed by local faith leaders. This study successfully included imams as they represent an important part of the social fabric for British Muslims. Capitalising on the strengths of religious institutions and developing long-term partnership between public health bodies and local Muslim faith leaders may open avenues for multiple other targeted and effective health-based interventions associated with perceived religious barriers.

Nevertheless, religious concerns were found not to be the sole barriers to OD registration in this study. Post-intervention, there remained a large number still unsure or against registration despite the assumption that it was permissible in Islam (17.0 and 3.6% respectively). This emphasises that aside from religious concerns other anxieties remain pertinent. Another unexpected finding was that amongst the 45 respondents already in possession of an OD card, 12 were unsure of OD’s religious permissibility, highlighting that for some religious uncertainty is not a barrier at all. Our educational interventions involved delivering some information on the technical processes and procedures of OD before delving into faith-based discussions. Our data suggests including such procedural and specialist information is important in motivating many Muslims. Healthcare professionals should not lose sight of this when conversing with Muslim patients and families.

With regards to our second hypothesis, our educational intervention created a significant increase in participant willingness to register. Overall, our intervention reduced uncertainty towards approval of OD. This is demonstrated in the statistically significant 25.8% increase in ‘yes’ responses to Question 2. This shift was generally found irrespective of age, gender or ethnic origin. The shift from ‘unsure’ before the intervention for questions 1 and 2 to more certainty and agreement with OD is demonstrated in Fig. 1. This large positive change suggests this is not an issue widely discussed amongst Muslim communities and that many of these communities remain in the pre-contemplation stage. Only 27.1% of respondents not carrying a donor card identified they had previously considered registering, and 53.6% indicated before the intervention that if OD was permissible they would be willing to register. The positive shift to 79% willing to register after the event, and the majority of the remaining respondents unsure rather than in opposition to registration, highlights the substantial potential for similar OD campaigns amongst UK Muslims (Ali et al., 2020a).

### Strengths, Limitations and Areas for Future Research

Strengths of this study include the large sample size and the use of a standard survey across multiple cities throughout the UK implying reproducibility and feasibility of future implementation. The primary limitation of this study is the lack of randomisation with the inherently open nature of the educational sessions, possibly resulting in a predisposition towards attendees with a high degree of uncertainty. Additionally, small sample sizes in certain subgroups (e.g. registered organ donors, White ethnicity) limit the generalisability of conclusions involving these groups. With multiple statistical analyses, the increase in familywise error rate across the reported statistical analyses was not controlled. Importantly, the translation of knowledge and attitudes to behaviour was not analysed in this study. Although there was positive movement post-intervention towards readiness to become an organ donor, and willingness to register matched views on permissibility, whether attendees later took action and signed a donor card (or did not opt out) is unclear and requires further follow-up. One study has previously shown only a small proportion of participants stating an intention to register actually do so at follow-up (Deedat et al., 2013).

There were some limitations in relation to study design and session content. Response rate could not be calculated as it was unfeasible to record the number of attendees. The programme was established and delivered by local volunteers; the lack of a fixed speaker panel produced a variability in quality of delivered content across locations. Though our questionnaire was self-administered, limiting the risk of responses being influenced by the interviewers, the fact that our questionnaire was not validated may have led to a misinterpretation of the results. Furthermore, the wording of the questionnaire was a limitation as a potential confounding factor. For example, Question 2 in Part A asked whether the participant would register for organ donation under the condition that it is religiously permissible, limiting the ability to extract independent conclusions on willingness to register.

Other possible improvements include broader demographic data collection to include data on education, income, employment, ownership of a driving license or years in the UK. Greater detail on perspectives is warranted such as willingness to accept an organ, views on live versus deceased donation and views on brain death.

Research on the opinions of Imams and local mosque leaders and the barriers to their involvement in health promotion and OD is lacking and is an area for future research–the only study on this issue included only three Muslim organisation leaders (Randhawa et al., 2010). Appropriate follow-up studies are essential to assess if these behavioural changes are actualised. Furthermore, it may be interesting to explore the specific barriers encountered by those who remain resistant to OD post-intervention and improve the content or delivery of these sessions. As this education programme is ongoing, we will be able to address the limitations mentioned previously, improve on the methodology and ensure these sessions are delivered effectively to the targeted communities.

## Conclusion

Our focussed local educational interventions produced a significant positive shift in opinion towards OD’s religious permissibility whilst reducing uncertainty and highlighted the presence of obstacles to registration aside from religious perception relevant to discussions at home as well as the clinical setting. Our study confirms the importance of direct grassroots work and the employment of members of the local ethnic and medical community to discuss these topics. Further work and follow up is needed to evaluate long-term efficacy. With a shortage of transplantable organs, growing Muslim communities and the UK transition to an opt-out system, there is an increasing need for the input of local community leaders, healthcare professionals and faith leaders to provide the information necessary to deal with medical, ethical, religious and cultural concerns regarding OD and enable the formulation of an informed decision.

## Data Availability

All data relevant to the study are included in the article or uploaded as online supplemental information.

## Appendix 1

Contents of the distributed questionnaire.
